# It all comes down to one: A rare case of a single coronary artery

**DOI:** 10.1002/ccr3.9353

**Published:** 2024-08-20

**Authors:** Konstantinos C. Christodoulou, Theodora M. Stougiannou, Anastasia Rigatou, Dimos Karangelis

**Affiliations:** ^1^ Department of Cardiothoracic Surgery University General Hospital of Alexandroupolis Alexandroupolis Greece; ^2^ Computed Tomography and MRI Department Sismanogleio General Hospital Athens Greece

**Keywords:** anatomical variation, atherosclerosis, coronary artery disease, coronary vessels

## Abstract

SCA is a rare congenital anomaly that, under certain conditions, can pose a life‐threatening risk to the individual. It is crucial to fully understand the entire course of the vessel and its anatomical relationships before developing a personalized treatment plan.

A 67‐year‐old male was referred to the outpatient clinic by his family doctor, due to unstable angina of 4 months and abnormal stress test. At presentation, the patient who was a heavy smoker for more than 40 years, with a medical history of dyslipidaemia (total cholesterol 320 mg/dL and low‐density lipoprotein 257 mg/dL), arterial hypertension (165/100 mmHg), and obesity (BMI = 33.9), was complaining about a chest pain of random onset and varying duration. Physical examination, apart from a grade 2 hypertension (165/100 mmHg), was unremarkable, including a normal electrocardiogram (ECG). Subsequently, an ECG gated coronary computed tomography angiography (CCTA) demonstrated coronary lesions in both the left anterior descending (LAD) and the right coronary arteries (RCA) (40%–60% and 20% stenosis of the medial LAD and RCA segments, respectively). Interestingly, an anomalous RCA was revealed; the vessel was originating from the left main coronary artery and not from the right sinus of Valsalva. Following an anterior to the pulmonary trunk pathway, the artery was coursing in the atrioventricular groove, following a pattern similar to the normal RCA (Figure 1). The patient was transferred to the cardiology department for further assessment.

**FIGURE 1:**
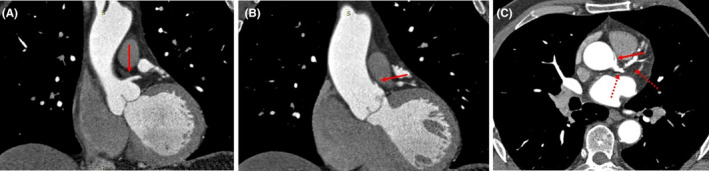
(A) Left main coronary artery (LMCA) originating from the left coronary sinus of Valsalva (red arrow). (B, C) Right coronary artery originating from the LMCA instead of the right coronary sinus of Valsalva (red arrows). LMCA is then branching into left anterior descending (LAD) and circumflex arteries (dotted arrows).

There have been numerous studies addressing anatomical variations of the coronary arteries, with a unanimous consensus yet to be reached. These variations, which affect the origin and/or course of the arteries, vary widely in their clinical presentation, spanning from asymptomatic to sudden death.^1^ In approximately 20% of the cases,^2^ they may be associated with life‐threatening haemodynamic changes, coronary artery disease (CAD) and sometimes with complex congenital heart diseases, however most of the cases are isolated.^1^

Single coronary artery (SCA), defined as the existence of a sole coronary ostium in the aortic sinuses of Valsalva, from which a single vessel is arising and supplying the entire myocardium, irrespective of its distribution and branching, could be deemed as such a variation. It is a very rare anomaly with a crude prevalence of 0.02%–0.06% in the general population, typically found incidentally during post mortem examinations.^2^ According to the three‐group classification proposed by Lipton et al., our case falls into the L‐IIA category, with a reported incidence of 0.009%.^2^

Given that the clinical significance of each anomaly is predominantly affected by the vessel's course rather than its point of origin, an LII type SCA passing anterior to the infundibulum is benign and does not impede perfusion. When SCA is implicated with CAD, a tailored therapeutic plan is mandated.^3^ Coronary angiography remains the gold standard for the evaluation of coronary arteries, with a high capability of identifying their anomalies. However, nowadays there is a trend towards the less invasive CCTA, which can delineate the complete anatomy of the coronary arteries, their variations, and any relations with adjacent structures.^2^

SCA is a rare congenital anomaly that, under certain conditions, can pose a life‐threatening risk to the individual. Unveiling the complete course of the vessel and understanding its anatomical relations is of utmost importance in devising an individualized treatment strategy.

## AUTHOR CONTRIBUTIONS

**Konstantinos C. Christodoulou:** Writing – original draft. **Theodora M. Stougiannou:** Investigation; writing – review and editing. **Anastasia Rigatou:** Data curation; validation. **Dimos Karangelis:** Supervision; writing – review and editing.

## FUNDING INFORMATION

None.

## CONFLICT OF INTEREST STATEMENT

The authors have no conflict of interest to declare.

## CONSENT

Written informed consent was obtained from the patient to publish this report in accordance with the journal's patient consent policy.

## Data Availability

Data may become available upon reasonable requests to the corresponding author.
